# Severe oral and intravenous insecticide mixture poisoning with diabetic ketoacidosis: a case report

**DOI:** 10.1186/1756-0500-7-485

**Published:** 2014-07-31

**Authors:** Narjis Badrane, Majda Askour, Kamal Berechid, Khalid Abidi, Tarek Dendane, Amine Ali Zeggwagh

**Affiliations:** 1 Centre Anti Poison et de Pharmacovigilance du Maroc, Rabat, Morocco; 2 Service de Réanimation Médicale-CHU Ibn Sina, Rabat-Faculté de Médecine et de Pharmacie de Rabat, Université Mohammed V Souissi, Rabat, Morocco

**Keywords:** Hyperglycemia, Pesticide, Poisoning, Ketosis

## Abstract

**Background:**

The widespread use of pesticides in public health protection and agricultural pest control has caused severe environmental pollution and health hazards, especially in developing countries, including cases of severe acute and chronic human poisoning. Diabetic ketoacidosis is an uncommon manifestation of acute pesticide poisoning. Suicidal pesticide poisoning by injection is also an unusual way to take poison. We report a severe pesticide mixture poisoning case with diabetic ketoacidosis in an adult with improved outcome after supportive treatment and large doses of atropine.

**Case presentation:**

A 30-year-old unmarried Moroccan Arab male with a previous history of active polysubstance abuse and behavior disorders had ingested and self injected intravenously into his forearm an unknown amount of a mixture of chlorpyrifos and cypermethrin. He developed muscarinic and nicotinic symptoms with hypothermia, inflammation in the site of the pesticide injection without necrosis. Red blood cell cholinesterase and plasma cholinesterase were very low (<10%). By day 3, the patient developed stroke with hypotension (80/50 mmHg) and tachycardia (143 pulses /min). Laboratory tests showed severe hyperglycemia (4.49 g/dL), hypokaliemia (2.4 mEq/L), glycosuria, ketonuria and low bicarbonate levels (12 mEq/L) with improvement after intensive medical treatment and treatment by atropine.

**Conclusion:**

Suicidal poisonings with self-injection of insecticide were rarely reported but could be associated with severe local and systemic complications. The oxidative stress caused by pyrethroids and organophosphates poisoning could explain the occurrence of hyperglycemia and ketoacidosis.

## Background

The trend toward the increased marketing of pesticide mixtures is likely to result in an increase in the prevalence of mixed toxicity [1]. Toxicity of pesticides may result from oral ingestion, inhalation, and absorption through the skin, but rarely through an injection [2]. One of the reported adverse effects in human exposure to pesticides is hyperglycemia. Organophosphates (OP) can influence body glucose homeostasis through several mechanisms including physiological stress, oxidative stress, inhibition of paraoxonase, nitrosative stress, pancreatitis, inhibition of cholinesterase, stimulation of the adrenal gland, and disturbance in the metabolism of liver tryptophan [3]. However, few studies have reported diabetic ketoacidosis with pesticide poisoning [4]. These cases have been reported in children and adolescents. We wish to present a severe pesticide mixture poisoning case with diabetic ketoacidosis in an adult with improved outcome after supportive treatment and large doses of atropine.

## Case presentation

A 30-year-old unmarried Moroccan Arab male was brought to the emergency within two hours of acute suicidal insecticide poisoning. He had ingested and self injected intravenously into his left forearm an unknown amount of an insecticide Synergy® (a mixture of chlorpyrifos 50% (CPF) and cypermethrin 5% (CM)).

He had previous history of active polysubstance abuse of benzodiazepines, alcohol, cannabis and recreational drugs used intravenously. His parents reported that he has habitually run away from home since his adolescence. He has also suffered from social isolation and religious delusions. The patient had never consulted a psychiatrist.

In the emergency room, vital signs revealed a pulse rate of 100 beats per minute, blood pressure of 170/100 mmHg, a respiratory rate of 25 breaths per minute and plenty of oral secretions. The patient was afebrile and had rhonchi all over his chest. Oxygen saturation was 80%, and Glasgow Coma Scale was 6/15. There were no fasciculations. The patient had also a miosis. He required ventilator support and he was admitted to the Medical Intensive Care Unit (MICU).

He developed, in few hours, hypothermia (34°C), bradychardia (35 beats per minute) with generalized fasciculations, tremor, excessive salivation, bronchial secretions and bronchospasm. Physical examination revealed hyperemia extending from the proximal third of the forearm to the axillary region with severe edema in the antecubital fossa without indurations or necrosis. The capillary refill time was normal and the pulses were present. Urine was discolored to a reddish brown. Investigations at admission to MICU, showed hyperglycemia (2.42 g/L), rhabdomyolysis (level of creatine kinase in the blood was 1188 UI/L) and low bicarbonate levels (16 mEq/L). Renal and liver functions and serum levels of sodium, potassium, calcium, and magnesium were normal. Blood picture showed leukocytosis. Screening for benzodiazepine, antiepileptic drugs, amphetamines, ethanol, cocaine, exstasy, tetrahydrocannabinol, morphine and its derivatives was negative. Red blood cell cholinesterase and plasma cholinesterase were very low (<10%). The chest X-ray and electrocardiogram were normal.

OP and pyrethroid (PYR) mixture poisoning was assumed on the basis of the medical interview, the compound identification made based on the container brought by the patient’s relatives, cholinergic syndrome associated with pyrethroid effects, tremor and excessive salivation supported by low plasma and red cell cholinesterase levels.

He was treated with, intravenous (IV) fluids, atropine, phenobarbital, IV sodium bicarbonate and passive external rewarming. Atropine (2 mg) was given every 10 minutes for four hours, followed by infusion at the rate of 2.5 mg per hour, and the dose was adjusted as per his clinical response. The use of phenobarbital was empirical because an electroencephalogram to look for subclinical seizures was not available. The patient was not treated with oxime because this antidote was not available.

At day 3, the patient developed stroke with hypotension (80/50 mmHg) and tachycardia (143 beats per minute). Laboratory tests showed severe hyperglycemia (4.49 g/dL), hypokaliemia (2.4 mEq/L), glycosuria, ketonuria, and low bicarbonate levels (12 mEq/L). Arterial blood gas analysis revealed pH 6.99, PaCO_2_ 73 mmHg, PaO_2_ 195 mmHg (FiO_2_ 70%), and HCO3ˉ 17.6 mEq/L, suggesting mixed acidosis. Cardiac enzymes and echocardiography were normal. Blood and urine were sterile. Procalcitonine was 1.90 ng/mL and C-reactive protein (CRP) was 2.70 mg/L. Amylasemia, lipasemia and glycosylated hemoglobin and abdominal ultrasound were normal. Treatment, including IV fluids, insulin infusion, parenteral potassium, sodium bicarbonate, adrenaline at the rate of 6 mg per hour and hydrocortisone-hemisuccinate was started. Treatment with atropine and supportive care was continued.

At day 5, he developed hyperthermia with chills. His chest X-ray was normal. The level of procalcitonin and CRP increased. *Streptococcus pneumoniae* was isolated from protected distal bronchial samples. Two bacteries, *Klebsiella pneumoniae* and *Staphylococcus hominis* were isolated from the blood. Empirical antibiotic therapy with ceftriaxone and gentamicin was started and modified to imipenem once bacteriological results became available. The glucose levels were normal and needed no further insulin therapy, and the acidosis was resolved at day 5.

Treatment with adrenaline was stopped on day 6. The patient required ventilator support for 7 days and atropine for 10 days. The patient received 700 mg as the total dose of atropine. Edema and inflammation in the left upper limb regressed without requiring surgery. Red blood cell cholinesterase and plasma cholinesterase were still very low (<10%). A psychiatric consultation conducted during hospitalization revealed a suicide attempt in the context of psychosis in the patient. He was discharged after 13 days to a medical department to continue the antibiotic therapy, clinical monitoring and to start the prescribed antipsychotic drugs. The serum cholinesterase has been recovered four weeks after poisoning.

## Discussion

There is an increased commercial interest in developing insecticide mixtures. The use of two active compounds in a mixture may provide rapid action and more residual effect than any of them if applied singly in sequence [1]. One of the most popular insecticide combinations is OP and PYR. The trend toward increased marketing of OP- PYR mixtures is likely to result in the creation of new patterns of mixed toxicity. The product used by our patient to attempt suicide was a mixture of CPF and CM.

Coexposure to CPF and CM inhibits the carboxylesterase mediated hydrolysis of CM, leading to an increased tissue concentration of this compound and a decreased urinary excretion of 3-phenoxybenzoic acid, the major metabolite of PYR [1]. Similarly, CPF oxon (the toxic metabolite of CPF) strongly and irreversibly inhibits CM hydrolysis [1]. These data could explain the severity of the clinical presentation of our patient. Indeed, the patient presented clinical signs related to the inhibition of acetylcholine esterase (ChE) by CPF but also prolonged tremor and hypersalivation because of the prolongation of the CM action by the inhibition of CM hydrolysis. Many experimental, clinical and in vitro studies have shown that CPF is associated with slower serum cholinesterase recovery and some animal studies confirmed that the inhibitory effect of CPF on ChE activity is not influenced by co-exposure to PYR [5]. The serum cholinesterase of our patient was very low (<10%) and recovered four weeks after poisoning.

A prospective study has showed that, in humans, OP poisoning causes an initial fall in body temperature, followed by a period of normal to high body temperature. However, there are factors such as infections and treatment that could alter thermoregulation in the patients with OP poisoning [6].

In our patient, it’s difficult to confirm that the cause of hyperthermia was the OP poisoning because of the presence of a major factor of confusion like nosocomial infection confirmed by the isolation of bacteria from protected distal bronchial samples and blood.

Hyperglycemia, after OP exposure, has been confirmed in animal studies [3]. Mechanisms of hyperglycemia demonstrated in these studies were the oxidative stress, inhibition of paroxanase, stimulation of adrenal glands and release of catecholamines, and the effect on metabolism of liver tryptophan [3].

Transient hyperglycemia and glycosuria are also found in severe OP poisoning [7].

The oxidative stress caused by PYR poisoning could explain the occurrence of hyperglycemia in PYR poisoning cases. An animal study showed that CM decreased the cellular antioxidant activity, altered marker enzyme activity and effected histopathological changes in the brain, heart, liver, kidney and testis of male rats [8].

To our knowledge, no study has shown the effect of pesticide mixtures on glucose metabolism. OP and PYR are responsible for an oxidative stress that could possibly explain the hyperglycemia induced by the mixture of OP and PYR. In fact, Wielgomas and Krechniak have shown that both CM and CPF administered as single compounds, or in combination, cause an impact on different free radical mediated parameters in wistar rats [1].

In our case, we have excluded diabetes. There was no patient history of diabetes, the glycosylated hemoglobin was normal and no other episode of hyperglycemia occurred during the hospitalization. The drugs administered to the patient before the installation of hyperglycemia, namely phenobarbital and atropine, don’t cause hyperglycemia as an adverse effect. We have excluded drug abuse because of the identification of illicit drugs and psychotropic drugs was negative, and because of the improvement of the patient after atropine therapy and supportive treatment.

Acute pancreatitis can occur with OP intoxication and may result in hyperglycemia [9]. We eliminated acute pancreatitis because of the normal amylase and lipase level and the normality of the abdominal ultrasound.

However, diabetic ketoacidosis is an uncommon manifestation of pesticide poisoning. The OP poisoning was the cause of the coma, hyperglycemia, glycosuria and keto-acidosis in a 3-year-old boy who was in contact with parathion [10]. OP intoxication can mimic diabetic ketoacidosis and its diagnosis may be delayed [4].

Akyildiz *et al.* have also reported a case of a 5-year-old girl with OP intoxication who presented as diabetic keto-acidosis [11]. Swaminathan *et al.* have discussed the case of a 15-year-old girl with diabetic ketoacidosis after an intentional overdose with OP pesticide [12]. Some adults with OP intoxication have presented with non-ketotic hyperglycemic coma [6]. However, no pesticide poisoning cases with diabetic ketoacidosis have been reported in adult patients.

The other originality of our observation was the unusual intravenous way used by the patient to attempt suicide. Suicidal poisonings with self-injection of insecticide have been rarely reported but could be associated with severe local and systemic complications [3,13]. Most patients had mental problems, such as depression, substance abuse, or both [3]. Our patient had a history of drug abuse and a diagnosis of psychosis was established in our department after a psychiatric consultation, which explains the patient’s choice of poisoning way.

Because of the severity of the intoxication, our patient required a large dose of atropine. A recent clinical study has shown that following atropinization, the maintenance dosage of atropine can be usually kept low at 0.005 mg h − 1 kg − 1 and doses of more than 0.06 mg h − 1 kg − 1 are only required when AChE is completely inhibited [14].

## Conclusion

To our knowledge, this is the first case of diabetic ketoacidosis caused by pesticide poisoning reported in adults. The oxidative stress caused by OP and PYR could play a role in the development of metabolic disturbances of glucose. The exact mechanisms of this action need further investigation.

Establishing the diagnosis of complications of pesticides poisoning is very important for adequate treatment and to improve the patient’s outcome.

## Consent

Written informed consent was obtained from the patient for publication of this Case Report. A copy of the written consent is available for review by the Editor-in-Chief of this journal.

## Abbreviations

OP: Organophosphates; MICU: Medical intensive care unit; IV: Intravenous; CRP: C-reactive protein; PYR: Pyrethroids; CM: Cypermethrin; CPF: Chlorpyrifos; ChE: Acetylcholine esterase.

## Competing interests

The authors declare that they have no competing interests.

## Authors’ contributions

NB, TD, KB, KA, MA, AAZ handled the case in the MICU. NB, TD, KA, KB, AAZ conceived the case report, and participated in its design. NB, TD, AAZ drafted the manuscript and sequence alignment of the report. NB, KB, MA, AAZ reviewed the literature. All authors read and approved the final manuscript.
